# Pneumatosis cystoides intestinalis and ketoacidosis in a diabetic patient: A case report and literature review

**DOI:** 10.1097/MD.0000000000043481

**Published:** 2025-07-18

**Authors:** Wenjie Zhou, Jie Dan, Mingjie Zhu, Ke Liu, Yonghong Wang

**Affiliations:** a Department of Gastrointestinal Surgery, The People’s Hospital of Leshan, Leshan, Sichuan Province, China.

**Keywords:** α glucosidase inhibitors, diabetes mellitus, ketoacidosis, misdiagnose, pneumatosis cystoides intestinalis

## Abstract

**Rationale:**

Pneumatosis cystoides intestinalis (PCI) is a rare disease characterized by gas accumulation in the intestinal wall, and is usually treated conservatively. Patients with diabetic ketoacidosis (DKA) sometimes present with severe abdominal pain as the first symptom, which may be misdiagnosed. We report a case of PCI in a patient with diabetes mellitus (DM) misdiagnosed as gastrointestinal perforation and underwent exploratory laparotomy.

**Patient concerns:**

A 41-year-old woman with a history of DM treated with miglitol, an α-glucosidase inhibitors (αGI), was admitted to the emergency department with severe abdominal pain. Computed tomography revealed thickening of the ascending colon wall and scattered free gas around it, and the possibility of tumor perforation was considered.

**Diagnosis:**

The patient was diagnosed with gastrointestinal perforation and DM but was revised to PCI and DKA after surgery.

**Interventions:**

The patient underwent exploratory laparotomy; however, no signs of digestive perforation were found. The patient developed DKA after surgery and received conservative treatment, including antibiotics, insulin, fluid support, oxygen therapy, and cessation of miglitol.

**Outcomes:**

Ketoacidosis was controlled, and the abdominal pain resolved with conservative treatment. She was discharged 16 days later and no longer required αGI therapy. She did not develop gastrointestinal symptoms or any signs of PCI on computed tomography imaging within 3 months.

**Lessons:**

PCI is a rare disease with great heterogeneity in etiology, treatment and prognosis and comorbidities like diabetes may increase the chances of misdiagnosis. Surgeons should pay attention to the patient’s medical history and examination and carefully identify the real disease that triggers the symptoms to avoid misdiagnosis.

## 1. Introduction

Pneumatosis cystoides intestinalis (PCI) is a rare disease characterized by multiple gas cysts in the serosa or submucosa of the digestive tract. It was first identified by Du Vernoi at autopsy in the 17th century and can occur throughout the entire digestive tract, especially the ascending colon, and is easily detectable on radiological examination. The etiology and pathogenesis of PCI remain unclear, and the actual incidence of PCI cannot be accurately calculated for patients are mostly asymptomatic or have mild symptoms. Patients with PCI alone are usually treated conservatively with a good prognosis. However, many patients may be complicated with multiple concomitant diseases and are prone to complications and some of them may be misdiagnosed with intestinal ischemia or gastrointestinal perforation thus being treated with surgical procedures.

In previous publications, PCI combined with diabetes mellitus (DM) was mostly caused by taking α-glucosidase inhibitors (αGI),^[1–8]^ and can be easily cured by ceasing αGI. Although PCI is easily misdiagnosed as gastrointestinal perforation on radiological imaging, surgeons will not easily resort to surgical treatment because the abdominal symptoms are mostly mild. We report a case of PCI combined with DM, in which the patient presented with severe abdominal pain, tachypnea, hyperpnea, and tachycardia, was misdiagnosed with gastrointestinal perforation, and then developed diabetic ketoacidosis (DKA) after surgery, which we suspected was present preoperatively. To the best of our knowledge, this may be the first reported case of PCI combined with a DKA. This case is discussed in depth, and the literature is reviewed as follows.

## 2. Case presentation

**Figure 1.**
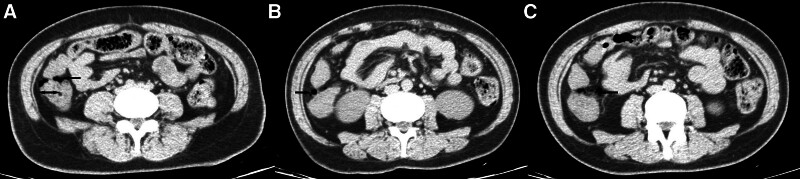
The black arrow indicates PCI. (A) Thickened bowel wall (bidirectional arrow) (B) PCI on the opposite side of the mesentery (C) PCI at the mesangial border like free gas black arrow. PCI = pneumatosis cystoides intestinalis.

A 41-year-old female was admitted to the hospital with a chief complaint of right lower abdominal pain for 3 days and sudden aggravation for 2 hours, accompanied by chills, tachypnea, hyperpnea, palpitations, nausea and vomiting. The patient was diagnosed with T2DM 8 months previously, and was well controlled with miglitol, empagliflozin and insulin. She presented with a distressed expression, severe pain in the right lower abdomen, pressing tenderness and suspected peritonitis on admission accompanied by a heart rate of 117 beats per minute and rapid, deep breathing of 24 beats per minute. Laboratory tests revealed the following leukocytes 12.56 × 10^12^/L, neutrophils 87.1%, high-sensitivity C-reactive protein 17.04 mg/L, blood glucose 11.97 mmol/L, sodium 133.9 mmol/L, and the rest of the laboratory tests did not show any obvious abnormalities. Computed tomography (CT) revealed thickening of the ascending colon wall and scattered free gas around it (Fig. 1), and the possibility of tumor perforation was considered. The patient was diagnosed with a gastrointestinal perforation and exploratory laparotomy was performed. However, we did not find any perforation lesions nor signs of infection but only swelling of the ascending colon on carefully examination of the entire digestive tract. Thus, the abdomen was closed after ileostomy according to the principle of damage control. The patient developed diabetic ketoacidosis (DKA) (arterial pH 7.26; β-hydroxybutyrate 6.14 mmol/L, urinary ketone+++) soon postoperatively and was improved through adequate fluid support and insulin therapy. αGI was discontinued after discharge and the patient did not develop gastrointestinal symptoms again or any signs of PCI on CT imaging within 3 months, thus stoma closure was performed.

## 3. Discussion and conclusion

The diagnosis was revised to PCI after a multidisciplinary discussion, which was considered to be associated with miglitol, a kind of αGI. Severe abdominal pain at the time of admission, and swelling of the intestinal wall, and extraintestinal gas on radiological imaging were the main reasons for the misdiagnosis. The patient presented with severe abdominal pain, shortness of breath, tachypnea, and tachycardia, which could not be explained by PCI alone and was highly suspected of DKA on admission. Although the patient was normoglycemic, he was taking a sodium/glucose co-transporter-2 inhibitor, empagliflozin, which has been widely reported to cause euglycemic DKA.^[9,10]^ The limitation of this case lies in the fact that we did not conduct arterial blood gas and ketone body tests before surgery. To our knowledge, this is the first case of hospitalization for both PCI and DKA. We conducted a search for relevant literature in PubMed, aiming to elaborate on the epidemiology, etiology, diagnosis, treatment methods, and prognosis of PCI.

### 3.1. Incidence

The incidence of PCI is generally considered low, and accurate statistics on its incidence are not available because many patients are asymptomatic. A cross-sectional survey showed that the detection rate of PCI using CT imaging in plateau areas of China reached 0.83%,^[11]^ and other studies have reported that plateau areas accounted for 66.9% of all reported cases.^[12]^ There are a few reports of PCI combined with DM, most of which are caused by αGI. We have not observed any report of misdiagnosis caused by PCI combined with DKA. We searched PUBMED with PCI and DM as key words, and found 21 English items with a total of 22 patients. The average age of these patients was 68 years, and 52.17% were female. The majority of these cases were from East Asia, with only 3 from Western countries. This is not necessarily due to racial differences, but the higher αGI use in Asians,^[6]^ because fat accounts for a larger proportion of caloric intake in western countries,^[13,14]^ which does not apply to the use of αGI. As we can see, 16 PCI cases were caused by αGI, and the duration of αGI use varied from 7 days to 14 years (Table 1).

**Table 1:** Diagnostic information of PCI patients with DM.

Author year	Age gender	Region	DM history	Symptoms and signs	PCI location	Other CT Feature	Blood tests	Concomitant disease and treatment
Dong Jin Park 2024^[15]^	57F	Korea	NA	None	Ileum	Necrotizing Enteritis	Normal	Metastatic Renal Cell Carcinoma; (Sunitinib)
Shinya Otsuka 2021^[1]^	59M	Japan	4 yr of Voglibose (0.9 mg daily); Insulin	None	Ascending To Descending Colon	Free Gas	Normal	Lung Transplantation; immunosuppressive Therapy (Tacrolimus and PSL)
Andrea Police 2020^[2]^	72	France	10 yr of Acarbose;	Abdominal pain and distention without peritonitis;	Sigmoid colon	Sigmoid Volvulus; Colonic Wall Thickened; Dolichocolon;	NA	NA
Divya S. Shetty 2020^[16]^	85F	Indian	NA	Abdominal distention; severe abdominal pain; constipation	Small intestine	PVG, SMVG Intestinal Obstruction	WBC 20;500/mm3 (NEU 86%)	NA
Minjia Wang 2020^[17]^	89M	China	NA	Fever And Abdominal Distention	Small Intestine and Colon	PVG SMVG	WBC 17,100/µL (NEU81.8%); CRP47.76 mg/L; PCT 5.99 ng/mL; PH 7.30	COPD; CAD Hypertension; Hypercholesterolemia
Yong Juan Wang 2018^[18]^	72F	China	10 yr of Acarbose; Insulin	Constipation; Diarrhea; Bloody Stool; Severe Abdominal Distension; Generalized Tenderness	Colon	NA.	Normal	
Eiji Suzuki 2017^[19]^	71F	Japan	Voglibose	Right Malar Rash; Saddle Nose; Right Abdominal Distension; No Tenderness	Small and Large Intestine	NA.	WBC 9;400/μL (NEU; 83.4%); HbA1c; 7.9% ESR 121 mm/h	Granulomatosis With Polyangiitis; (PSL)
	70F	Japan	10 yr of Voglibose	Severe Back Pain; Body Temperature Was Slightly High	Colon	Free Gas	WBC 17;700/μL (NEU; 88.5%); HbA1c;5.5%; CRP; 14.63 mg/dl; ESR; 38 mm/h	Rheumatoid Arthritis (PSL)
Yen-Hsiu Liao 2017^[3]^	50M	Taiwan	1 yr of Acarbose (150 mg daily)	Dull Abdominal Pain, Fever; Diarrhea; Green Stool.	Colon	NA.		NA.
Dorota Ksiadzyna 2016^[20]^	64M	Netherlands	8 yr of Acarbose (150 mg daily) Metformin (1.5 g daily)	Diarrhea; Flatulence; Diffuse and Mild Abdominal Pain	Cecum And Splenic Flexure Colon	NA.	Normal	NA.
Hiroaik Makiyami 2014^[21]^	80F	Japan	Voglibose (0.6 mg daily)	Abdominal Distention; Discomfort; Tenderness; Vomiting; No Peritonitis	Ileum	PVG	WBC 15,400/μL (NEU;82.8%)	NA.
Shunsuke Tanabe 2013^[4]^	80F	Japan	α-GI	Constipation; Abdominal Distention; Pain; Mild Tenderness; No Peritonitis	Intestinal Tract Wall	Free Gas	Normal	Hypertension; Hemiplegia
Yasuhiro Shimojima 2011^[22]^	48M	Japan	6 wk of Voglibose, Glimepiride	Tenderness; Without Peritonitis	Colon; Especially Ascending Colon		Normal	Systemic Lupus Erythematosus; (PSL)
Kuniyuli Kojima 2010^[5]^	58M	Japan	2 yr of Miglitol (150 mg daily)	Abdominal Pain;Rectal Bleeding	Ileocecal and Ascending Colon	Thickening Bowel Walls, Free Gas	Normal	NA.
Hideto Suzuki 2009^[23]^	67F	Japan	Diet therapy	Nausea; Abdominal Pain; Mild Tenderness; Hypopnea of Bowel Sounds;	Colon	NA.	WBC (10;700/ul)	NA
Tatsuhiro Tsujimoto 2008^[6]^	69M	Japan	3 yr of α GI	Abdominal Distension; Constipation; Rectal Bleeding;	Sigmoid Colon;	Free Gas	HbA1c 6.0%;	Myasthenia Gravis (PSL)
Yoshitaka Maeda 2007^[24]^	72F	Japan	3 yr of Voglibose (0.9 mg daily); insulin	Fever; Abdominal Pain; Transient Unconsciousness; Abdominal Tenderness;	NA	Intestinal Lumen Narrowed	WBC 11,500/μL	Nephrotic Syndrome; Immunosuppressive Therapy (PSL And Mizoribine)
Akiko Hismoto 2006^[7]^	56F	Japan	7 d of Voglibose (600 mg daily)	None	Ascending Colon;	NA.	Normal	Interstitial Pneumonitis; Prednisone
K. Nakamura 2003^[25]^	56M	Japan	NA	None	Cecum To Transverse Colon	NA	Normal	Renal Transplant; Immunosuppressive (Tacrolimus; PSL; Mizoribine; 15-Deoxyspergualin)
Yasushi Azami 2000^[26]^	87F	Japan	14 yr of Glibenclamide (5 mg daily), Acarbose (150 mg daily);	Abdominal Distension; Loss of Appetite; No Peritonitis	Small Intestine	Gaseous Distension of Small Intestine	Normal	Hypothyroidism Paralytic Ileus.
Hayakawa T. 1999^[8]^	64F	Japan	20 yr of insulin, 2 mo of voglibose (0.6 mg/d)	Abdominal Distention	Ascending to Proximal Transverse Colon	NA.	HbA1c 7.0%	NA.
Bonnell H 1982^[27]^	72M	American	17 yr of Insulin	NA.	NA.	NA.	NA.	Pulmonary edema, renal failure, osteomyelitis

CRP = C-reaction protein, CT = computed tomography, DM = diabetes mellitus, ESR = erythrocyte sedimentation rate, NA = not applicable, NEU = neutrophil, PCI = pneumatosis cystoides intestinalis, PSl = prednisolone, PVG = portal vein gas, SMVG = superior mesenteric vein gas, TPN = total parenteral nutrition, WBC = white blood cell.

### 3.2. Etiology

The cause of PCI is unknown and may be associated with a number of comorbidities, traumatic procedures or medication use, such as: Respiratory diseases,^[28,29]^ connective tissue diseases,^[30,31]^ malignancies,^[15,32]^ Surgical complications,^[33,34]^ gastrointestinal dysfunction,^[35]^ Immunosuppressive agents^[36,37]^ and so on. In the past decades, some theories about its mechanism have been gradually formed, with the most famous being the mechanics theory and microbial fermentation theory. When the physical and immune barriers of the intestine are damaged for various reasons, increased intestinal pressure or lung diseases may lead to the diffusion of gas into the submucosa and serosal layer, where bacteria colonize and ferment to convert carbohydrates into hydrogen to form cysts.^[38]^ However, no single theory perfectly explains the pathophysiology of PCI. Several studies have shown that αGI administration is the main cause of PCI in patients with DM.^[18–20,22,24,26]^ Patients with DM tend to use αGI more frequently in Asian countries. This may be due to the eating habits of the population, which may affect the composition of their intestinal microbiota, and the fermentation process of this microbiota may play an important role in the occurrence of PCI. Therefore, we believe that a nutritionist may also play an important role in managing these cases, as they do in the management of cancer patients.^[39]^ However, 27% of the patients did not have a clear history of αGI, and approximately half of the patients had other underlying diseases and drug use (Table 1). Therefore, it is necessary to pay more attention to diabetes patients with no history of αGI administration when PCI occurs, as the treatment and prognosis may be quite different.

### 3.3. Diagnosis

Depending on the comorbidities, patients undergoing PCI may have different clinical presentations. Patients undergoing PCI alone or due to αGI are usually asymptomatic or have only mild nonspecific gastrointestinal symptoms, manifesting as abdominal distension, abdominal pain, nausea, vomiting, diarrhea, constipation, rectal bleeding, and weight loss according to the distribution in different intestines. Laboratory tests results are usually nonspecific, and the markers of infection are mostly normal or mildly elevated. However, patients may also present with severe gastrointestinal or systemic symptoms; the patient we report is a particular example, although we suspect that it was caused by DKA. Particularly, attention should be paid to the complications of PCI, such as volvulus,^[2,40]^ intestinal ischemia,^[16]^ intestinal obstruction,^[41,42]^ intussusception^[43–45]^ and uncontrolled gastrointestinal bleeding,^[46,47]^ which may require surgical intervention and are potentially life-threatening.

CT imaging is important in the diagnosis of PCI. Multiple cystic air density shadows of different sizes were observed in the intestinal wall, some of which were connected in strings, protruding from the inside and outside of the intestinal lumen. No obvious exudation was observed around the cysts. These lesions can be distributed throughout the digestive tract, but mainly in the intestines, especially in the colon. It should be noted that PCI can also show other special imaging signs, such as extraluminal free air,^[1]^ intestinal wall thickening,^[2]^ and portal vein gas (PVG),^[21,48]^ which are easily misdiagnosed as gastrointestinal perforation, intestinal necrosis, or tumor. Among the 22 patients of PCI with DM, 5 patients presented with free abdominal gas, 3 with PVG and 2 with bowel wall thickening (Table 1). A cross-sectional study in Tibet have shown that the incidence of PCI complicated with free abdominal gas is up to 60.36%,^[11]^ which may lead to a high rate of misdiagnosis and most patients do not have severe symptoms. Therefore, CT imaging should not be over-relied upon to assess the condition of patients with PCI. Careful history-taking and physical examination are the basis for preventing misdiagnosis. In addition, endoscopy and plain abdominal radiography are diagnostic methods for PCI but appear to be less sensitive or convenient when compared with CT imaging.

### 3.4. Treatment

Most patients with PCI alone improved with conservative treatment. Discontinuation of αGI therapy in patients with DM is extremely important. Other treatments include fasting, rehydration, antibiotics, endoscopic therapy, hyperbaric oxygen therapy, and probiotics, the effectiveness of which is controversial (Table 2), such as oxygen therapy.^[25,38]^ Although most patients can improve after the above treatments, some patients still require surgery even emergency surgery. A systematic analysis reported that the operation rate in China was 40.6% in 10 years,^[12]^ among which many were nontherapeutic or misdiagnosed operations with a rate of about 15% in a report from the University of Virginia.^[49]^ Since the probability of abdominal free gas after subserosal PCI rupture is 60.36%,^[12]^ it must be strictly differentiated from gastrointestinal perforation and surgical exploration is recommended for patients with severe symptoms, high white blood cell count, and extensive abdominal exudation on imaging. Among the 22 patients with PCI and DM, 3 underwent surgery, 2 of whom underwent intestinal resection due to sigmoid colon torsion and small bowel ischemia caused by PCI, respectively, and the other was suspected of intestinal necrosis due to hepatic PVG on CT imaging and underwent laparotomy, but no intestinal abnormalities were found.^[21]^ In our view, surgery is performed to address the comorbidities or complications of PCI rather than PCI itself. It can be considered for PCI patients with intestinal volvulus, intestinal obstruction, necrosis or uncontrolled gastrointestinal bleeding. Laparoscopic exploration may also be considered when vital signs are stable. However, emergency surgery is not always necessary.

**Table 2:** Treatment and prognosis of PCI patients with DM.

Author/year	Conservative treatment	Surgery	Recovery time and evaluation method	Poor prognosis
Park 2024^[15]^	TPN*†‡, ceasing oral diabetes drugs		3 mo (CT)	
Otsuka 2021^[1]^	Bowel rest†‡§		11 d (X-ray)	
Police 2020^[2]^	–	Sigmoidectomy	1 mo (symptoms)	–
Shetty 2020^[16]^	–	Enterectomy	–	Died
Wang 2020^[17]^	Palliative therapy	–	–	Died
Wang 2018^[18]^	Endoscopic*§ treatment; probiotics	–	1 mo (symptoms) 6 mo (colonoscopy)	–
Suzuki 2017^[19]^	Properistaltic agents§	–	1 mo (CT)	–
	* §	–	2 wk (CT and laboratory)	–
Hsiu Liao 2017^[3]^	§	–	NA	–
Ksiadzyna 2016^[20]^	§	–	4 wk (symptoms, CT)	–
Makiyami 2014^[21]^	§	Laparotomy	NA (symptoms)	–
Tanabe 2013^[4]^	* †	–	1 d (symptom), 2 d (CT)	–
Shimojima 2011^[22]^	Ceasing† glimepiride panthenol and dinoprost	–	1 wk (X-ray free air disappeared) 19 wk (X-ray PCI disappeared)	–
Kojima 2010^[5]^	† §	–	12 d (colonoscopy);	–
Suzuki 2009^[23]^	Laxative; enema	–	–	Died
Tsujimoto 2008^[6]^	† §	–	2 wk (X-ray); 3 mo (colonoscopy)	–
Maeda 2007^[24]^	Ventilator† therapy; hemofiltration	–	Rapid deterioration and eventually improved	–
Hismoto 2006^[7]^	† ‡	–	1 wk (symptoms)	–
Nakamura 2003^[25]^	‡	–	–	No efficacy
Azami 2000^[26]^	† §	–	5 d (symptoms and CT)	
Hayakawa 1999^[8]^	† §	–	4 d (X-ray)	
Bonnell 1982^[27]^	–	–	–	Died

CT = computed tomography, DM = diabetes mellitus, PCI = pneumatosis cystoides intestinalis, TPN = total parenteral nutrition.

* Antibiotics.

† Fasting and fluid support.

‡ Oxygen therapy.

§ Ceasing α-GI

### 3.5. Prognosis

The prognosis of PCI patients with DM is generally good. A prediction model for intestinal ischemia in PCI patients also suggested that patients with DM were less likely to undergo surgical intervention, which may be due to αGI induction in most PCI patients with DM. However, we retrieved 4 PCI patients with DM who eventually died (Table 2), and all of them were due to comorbidities,^[16,17,23,27]^ which may mean that the prognosis of patients depends more on comorbidities or complications than on PCI itself. Patients with PCI are easily misdiagnosed and treated with nontherapeutic procedures, which may aggravate comorbidities and lead to a poor prognosis. According to an appellate study, the rate of noncurative surgery is as high as 15%.

The limitations of this review lie in its focus on the analysis of PCI combined with DM. The cited literature is mostly case reports, and some of the publications were from a long time ago, resulting in insufficient data and information. Although we made every effort to conduct a thorough review of PCI, there may still be some inadequacies that prevent readers from gaining a clear understanding of PCI.

In conclusion, PCI is a rare disease with significant heterogeneity in etiology, treatment and prognosis. It is easily misdiagnosed and its pathophysiology is still poorly understood. In patients with diabetes, the use of αGI is the most common cause of PCI, and the prognosis is good with conservative treatment. Surgery is mainly aimed at comorbidities or complications, and surgeons should pay attention to the patient’s medical history and examination and carefully identify the real disease that triggers the symptoms to avoid misdiagnosis and mistreatment.

## Author contributions

**Conceptualization:** Wenjie Zhou.

**Investigation:** Wenjie Zhou.

**Supervision:** Yonghong Wang.

**Visualization:** Wenjie Zhou, Yonghong Wang.

**Writing – original draft:** Wenjie Zhou, Jie Dan, Mingjie Zhu, Yonghong Wang.

**Writing – review & editing:** Wenjie Zhou, Jie Dan, Mingjie Zhu, Ke Liu, Yonghong Wang.
